# Multiresponsive Soft Actuators Based on a Thermoresponsive Hydrogel and Embedded Laser-Induced Graphene

**DOI:** 10.1021/acsapm.0c01385

**Published:** 2021-03-09

**Authors:** Alexander Dallinger, Paul Kindlhofer, Francesco Greco, Anna Maria Coclite

**Affiliations:** Institute of Solid State Physics, NAWI Graz, Graz University of Technology, Graz 8010, Austria

**Keywords:** multiresponsive, laser-induced graphene, soft actuator, smart hydrogel, pNVCL, hygromorphic

## Abstract

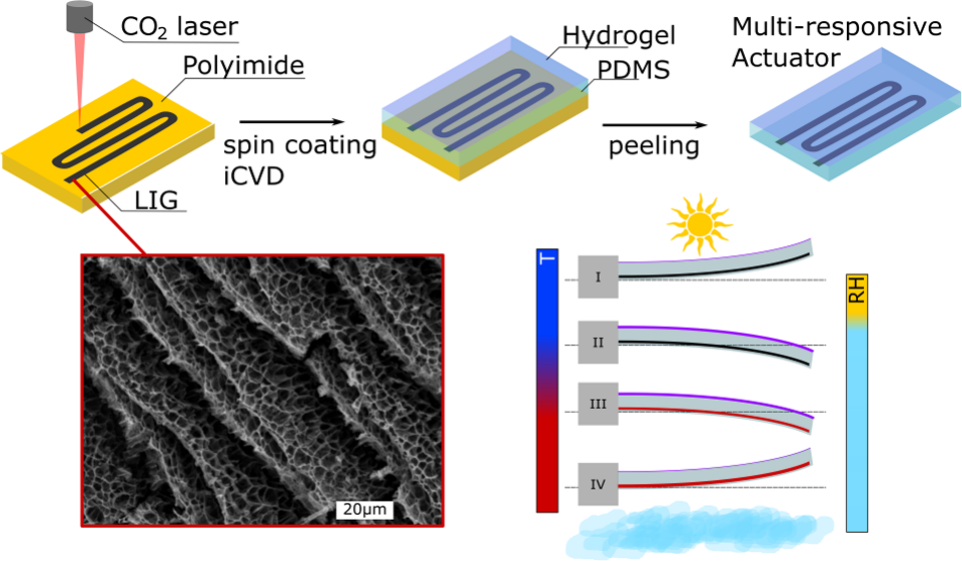

The method of converting insulating polymers into conducting 3D porous graphene structures, so-called laser-induced graphene (LIG) with a commercially available CO_2_ laser engraving system in an ambient atmosphere, resulted in several applications in sensing, actuation, and energy. In this paper, we demonstrate a combination of LIG and a smart hydrogel (poly(*N*-vinylcaprolactam)—pNVCL) for multiresponsive actuation in a humid environment. Initiated chemical vapor deposition (iCVD) was used to deposit a thin layer of the smart hydrogel onto a matrix of poly(dimethylsiloxane) (PDMS) and embedded LIG tracks. An intriguing property of smart hydrogels, such as pNVCL, is that the change of an external stimulus (temperature, pH, magnetic/electric fields) induces a reversible phase transition from a swollen to a collapsed state. While the active smart hydrogel layer had a thickness of only 300 nm (compared to the 500 times thicker actuator matrix), it was possible to induce a reversible bending of over 30° in the humid environment triggered by Joule heating. The properties of each material were investigated by means of scanning electron microscopy (SEM), Raman spectroscopy, tensile testing, and ellipsometry. The actuation performances of single-responsive versions were investigated by creating a thermoresponsive PDMS/LIG actuator and a humidity-responsive PDMS/pNVCL actuator. These results were used to tune the properties of the multiresponsive PDMS/LIG/pNVCL actuator. Furthermore, its self-sensing capabilities were investigated. By getting a feedback from the piezoresistive change of the PMDS/LIG composite, the bending angle could be tracked by measuring the change in resistance. To highlight the possibilities of the processing techniques and the combination of materials, a demonstrator in the shape of an octopus with four independently controllable arms was developed.

## 1 Introduction

Actuators turn an input signal (stimulus) into mechanical motion. Soft actuators, which change their size and shape by an applied stimulus (electrical fields, heat, pH), are based on polymers.^1^ Such actuators are becoming more important in a wide range of fields like artificial muscles for (soft) robotics or biomimetics. Soft actuators based on different materials (dielectric elastomers,^2,3^ paper,^4−6^ carbon materials,^4−6^ thermoresponsive^7^ and conducting polymers^8−10^) were shown in the past. New combinations of materials and stimuli can enable actuators to function in new ways and environments. In this contribution, a controllable and multiresponsive actuator is demonstrated. The basic idea was to combine the tunable temperature response of a smart hydrogel with the possibility of inducing localized heating, accomplished with a Joule heater based on a new variant of laser-induced graphene (LIG) embedded in a composite material structure.

The embedded LIG tracks not only allow the local control of actuation but can also be used for feedback on the actuation. The piezoresistive change induced by the bending could be measured and related to the bending angle. Control and self-sensing are indeed very important features in any actuation application, including biomimetic soft actuators for locomotion and grippers among others.^11^

Laser scribing of LIG is a method for creating conductive patterns from insulating polymer precursors. LIG is obtained by laser scribing polyimide (PI) with a commercial CO_2_ laser in an ambient atmosphere. This makes it possible to create conductive patterns with customized design on top of the insulating PI in a scalable fashion. This was first investigated by the group of James M. Tour^12^ and attracted some attention over the last few years. The PI is converted into a complex 3D porous graphene structure by a photothermal process, which is induced when a certain threshold of laser fluence is reached.^13^ The PI locally attains temperatures >2400 K that causes a pyrolysis of the precursor, resulting in the release of volatile gases such as CO and H_2_, and a conversion of the sp^3^ carbons into sp^2^ carbons arranged into a defective graphene structure. This leads to the porous structure and unique morphology of LIG.^14^ Because the heating and cooling happen very fast, 5-, 6-, and 7-membered rings of carbon are formed, which distinguishes LIG from traditional graphene. Moreover, the C, N, and O composition of LIG surfaces can be tuned with processing parameters, leading to different surface properties.^15^ Among the many different applications, LIG has been tested for use in thermoresponsive actuators.^16,17^ The conductive LIG patterns were used as Joule heating elements to induce deformation/actuation based on the mismatch of the coefficient of thermal expansion between the LIG scribed on PI and an encapsulating polymer matrix. Because the coefficient of thermal expansion of PI is relatively small (20–60 ppm/K), normally a second material (matrix) with a larger coefficient is needed for the thermoresponsive actuation (e.g., PDMS^16^ or PVDF^17^). On the other hand, smart hydrogels are increasingly used for soft actuators. The hydrogels can swell up to several times their original thickness (i.e., thickness in the dry state) if immersed in water or exposed to humidity. The change of an external stimulus (temperature, pH, magnetic/electric fields, depending on the specific functionalization) induces a reversible phase transition, where the hydrogel changes from a swollen state to a collapsed state. In this study, a temperature- and humidity-responsive hydrogel is investigated for this purpose. Initiated chemical vapor deposition (iCVD) of the smart hydrogel is chosen as the polymerization technique because it allows conformal coating, in the nanometer regime, on almost any surface, as well as the retention of the functional groups of the used monomers, stemming from the fact that it operates at relatively low temperatures.^18^ Recent investigations of poly(*N*-vinylcaprolactam) (pNVCL) by Muralter et al. showed that it is possible to create thermoresponsive actuators based on PDMS and very thin pNVCL layers.^19^ It was possible to tune the phase transition temperature, the so-called lower critical solution temperature (LCST) at which the polymer network passes from a swollen state to a collapsed state, by changing the fraction of the cross-linking agent, namely, di(ethylene glycol) divinyl ether (DEGDVE). A proof-of-concept actuator was demonstrated, which reacted to a change of humidity and temperature by changing its form (open/closed flower petals).^19^ The possibility of freely patterning conductive LIG shapes and an easy transfer onto a soft actuation matrix in combination with iCVD, a deposition method that allows delicate materials such as soft polymers to be coated with smart hydrogels (thermo-, humidity-, or light-responsive), opens up the possibility for a new combination of materials for actuators. Furthermore, this approach would allow scaling up to large-scale applications because of the used processing techniques.

In this work, the fabrication and characterization of a poly(dimethylsiloxane) (PDMS) actuator with embedded conductive tracks from LIG and a thermoresponsive hydrogel coating (pNVCL) is shown. The LIG is converted by laser-induced pyrolysis of PI, a common material for flexible electronics. By changing the laser fluence H, a new variant of LIG was created. A unique feature of this variant is that it is lifted off from the PI substrate, and this makes it possible to completely embed it in PDMS by spin coating. LIG is investigated through scanning electron microscopy (SEM) imaging, and its composition is assessed by means of Raman spectroscopy. The determination of the thickness of the hydrogels and the LCST, as well as the in situ swelling experiments, is performed with spectroscopic ellipsometry. Soft actuators based on PDMS/pNVCL, PDMS/LIG, and PDMS/LIG/pNVCL are characterized by means of bending experiments under controlled conditions. Recorded movies of the experiments are used to extract the bending angle and the resulting curvature for each type of actuator depending on the relative humidity and heating current/temperature.

## 2 Results and Discussion

**Figure 1:**
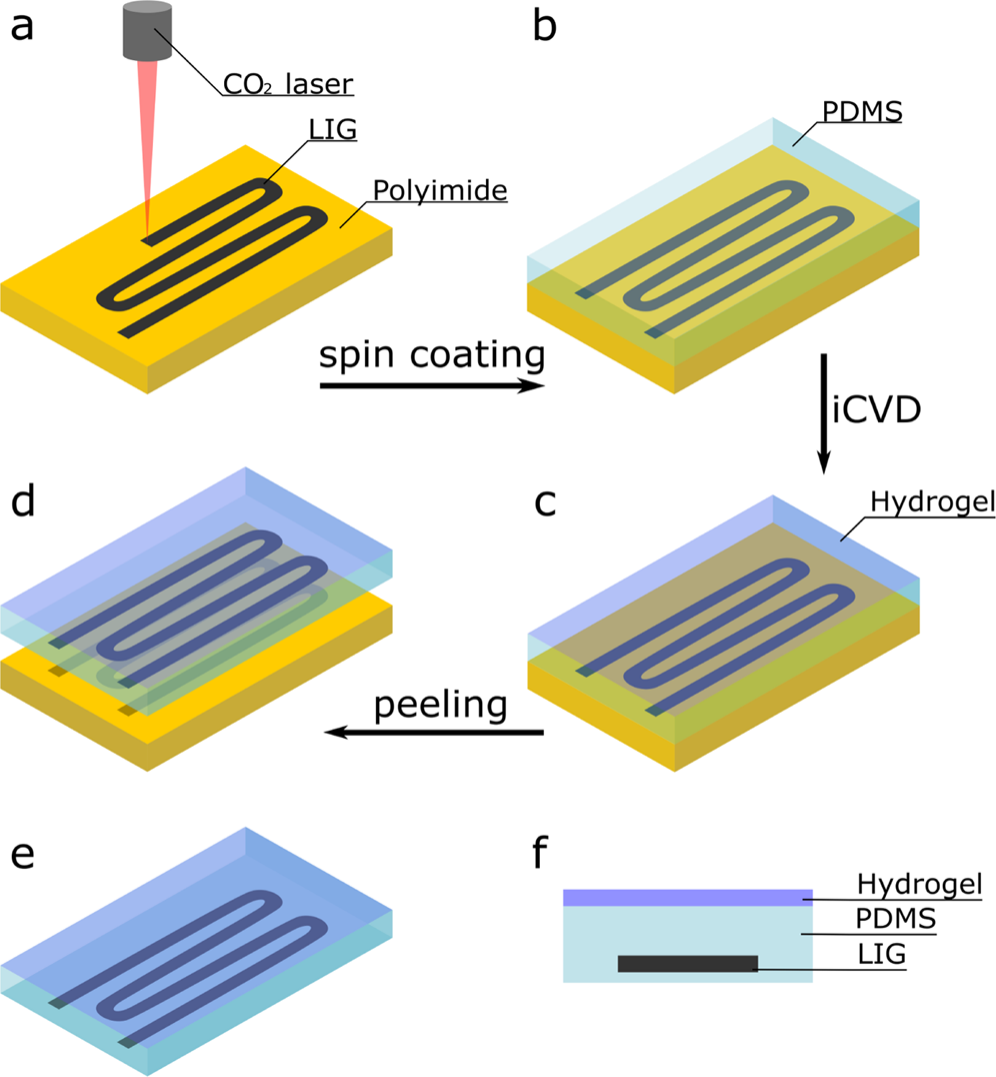
Schematic for fabrication of multiresponsive actuators: (a) laser scribing polyimide with patterns; (b) spin coating of PDMS on top of the scribed PI with LIG tracks; (c) iCVD deposition of the pNVCL hydrogel; (d) peeling of the actuator from the PI substrate; (e) finished multiresponsive actuator; and (f) cross section of the actuator consisting of PDMS, embedded LIG, and the smart hydrogel.

The steps that are required to produce a multiresponsive soft actuator are illustrated and described in Figure 1. Figure 1a shows the conversion of PI into LIG by laser scribing patterns with a commercial CO_2_ laser. Next, spin coating of the PI and conductive LIG patterns with PDMS to obtain a soft actuator matrix (Figure 1b) is followed by iCVD deposition of the thermoresponsive hydrogel pNVCL (Figure 1c). The last step is to peel the actuator from the PI substrate (Figure 1d) to obtain a soft and stretchable actuator (Figure 1e). A schematic cross section of the soft actuator consisting of the PDMS matrix, embedded LIG, and deposited pNVCL is illustrated in Figure 1f.

### 2.1 Characterization of Materials

**Figure 2:**
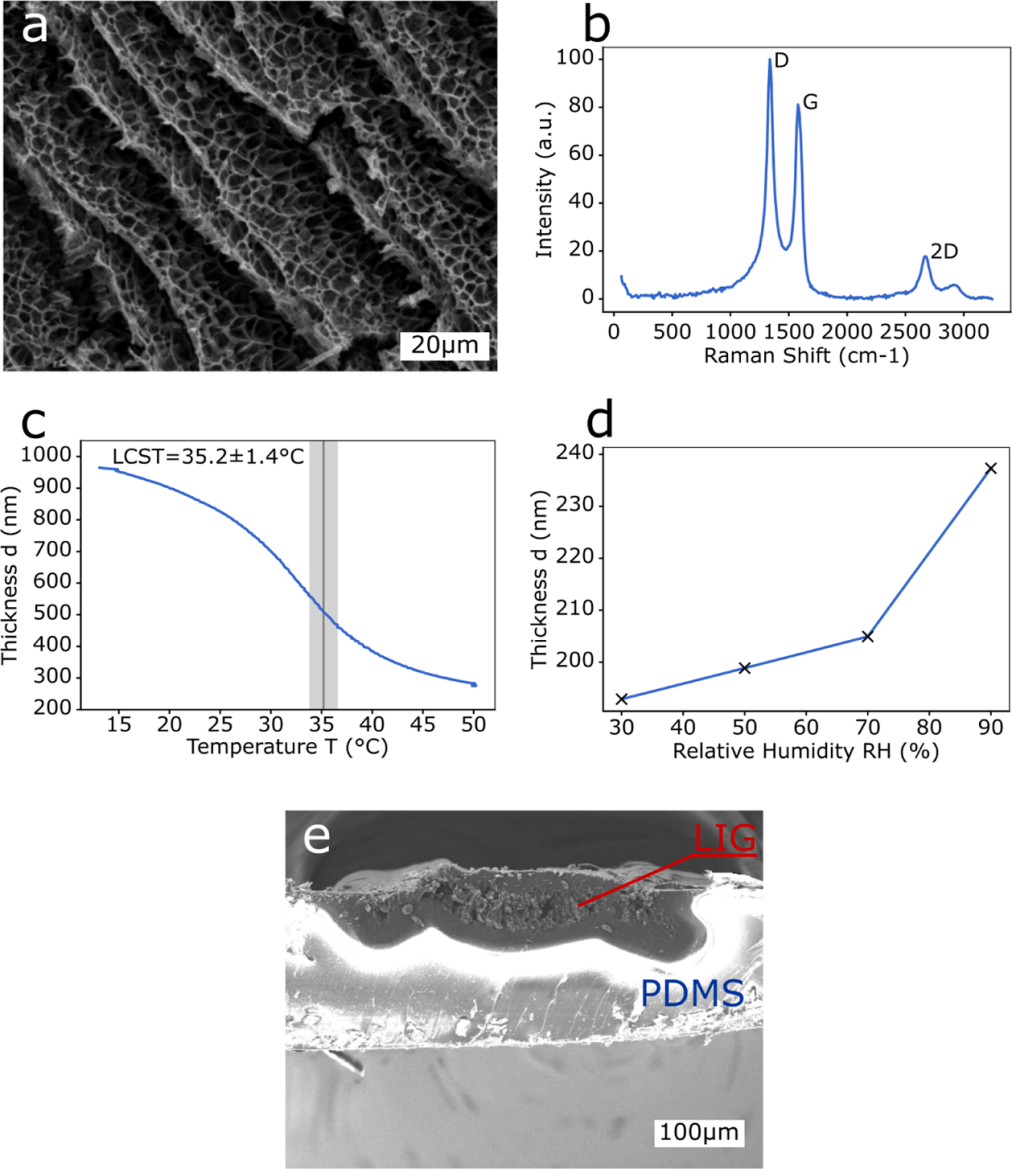
(a) SEM image of LIG-LO; (b) Raman spectrum of LIG-LO showing characteristic D, G, and 2D bands; (c) change of thickness *d* with temperature *T* for evaluation of the LCST; (d) swelling of the hydrogel in a humid environment as a function of relative humidity RH; and (e) SEM image of a cross section of LIG-LO embedded in PDMS.

The parameters for laser scribing PI were tuned to obtain a new species of LIG; this variant stood out because the resulting porous graphene features, unlike other variants obtained so far (fiber, porous),^20^ were not attached to the PI but instead were lifted off from the surface during scribing. For this reason, we refer to it as LIG-lift off (LIG-LO). While the laser fluence for the LIG-LO setting was rather low (*H* = 10 J/cm^2^), the big overlap (high ID and PPI settings) resulted in multiple lasering of the scribed LIG and in its lifting off. This allowed for a very easy transfer onto different soft substrates and the embedding into a liquid precursor (like PDMS). Figure 2 shows some properties of the used materials that are important for the application. In Figure 2a, an SEM image illustrates the morphology of LIG-LO, showing a very porous network of multilayered graphene slabs. The Raman spectrum for this LIG variant (Figure 2b) displays the typical three features of LIG: the D band at 1339 cm^–1^, the G band at 1583 cm^–1^, and the 2D band at around 2676 cm^–1^. While the D band is associated with disorder and defects, the *I*_D_/*I*_G_ intensity ratio describes the crystallinity of the LIG.^21^ With a ratio of 1.11, this LIG variant had a lower degree of crystallinity compared to other LIG variants investigated in a previous contribution.^20^ A full table of all features can be found in Supporting Information Table S1.

The properties of the hydrogel were investigated by means of swelling experiments performed with an ellipsometer equipped with a heated liquid cell and a variable temperature stage. The starting point for the deposition conditions was the investigations by Muralter et al.,^19,22^ who studied the change in LCST and the swelling behavior depending on the incorporated cross-linker fraction and the deposition conditions. The change in the thickness of the film with temperature is shown in Figure 2c; the swelling ratio *d*_*T*=15_/*d*_*T*=55_ was 3.45. The LCST (point of inflection) was measured to be at *T*_LCST_ = (35.2 ± 1.4) °C. Comparing this, the swelling with humidity at *T* = 25 °C is plotted in Figure 2d; the swelling ratio *d*_RH=90_/*d*_RH=30_ was 1.25. The different swelling ratios in Figure 2c,d can be attributed to the fact that there is a significant increase in the thickness of the hydrogel above RH = 90%.^19^ A detailed plot of thickness over time at different humidity levels can be found in the Supporting Information (Figure S1). The thermal stability of the hydrogel film was evaluated and showed no signs of degradation up to *T* = 150 °C (Figure S2). The PDMS was characterized and a thickness of *d*_PDMS_ = (140 ± 30) μm was found by measuring the cross section under an optical microscope. The Young’s modulus was measured to be *E*_PDMS_ = (2.5 ± 0.3) MPa (stress–strain curve in Figure S3, Supporting Information) with a custom tensile testing setup described already elsewhere.^20^ SEM imaging of a cross section of the PDMS/LIG composite (Figure 2e) shows that the LIG with a thickness of around *d*_LIG_ = 40 μm is fully embedded in the PDMS.

**Figure 3:**
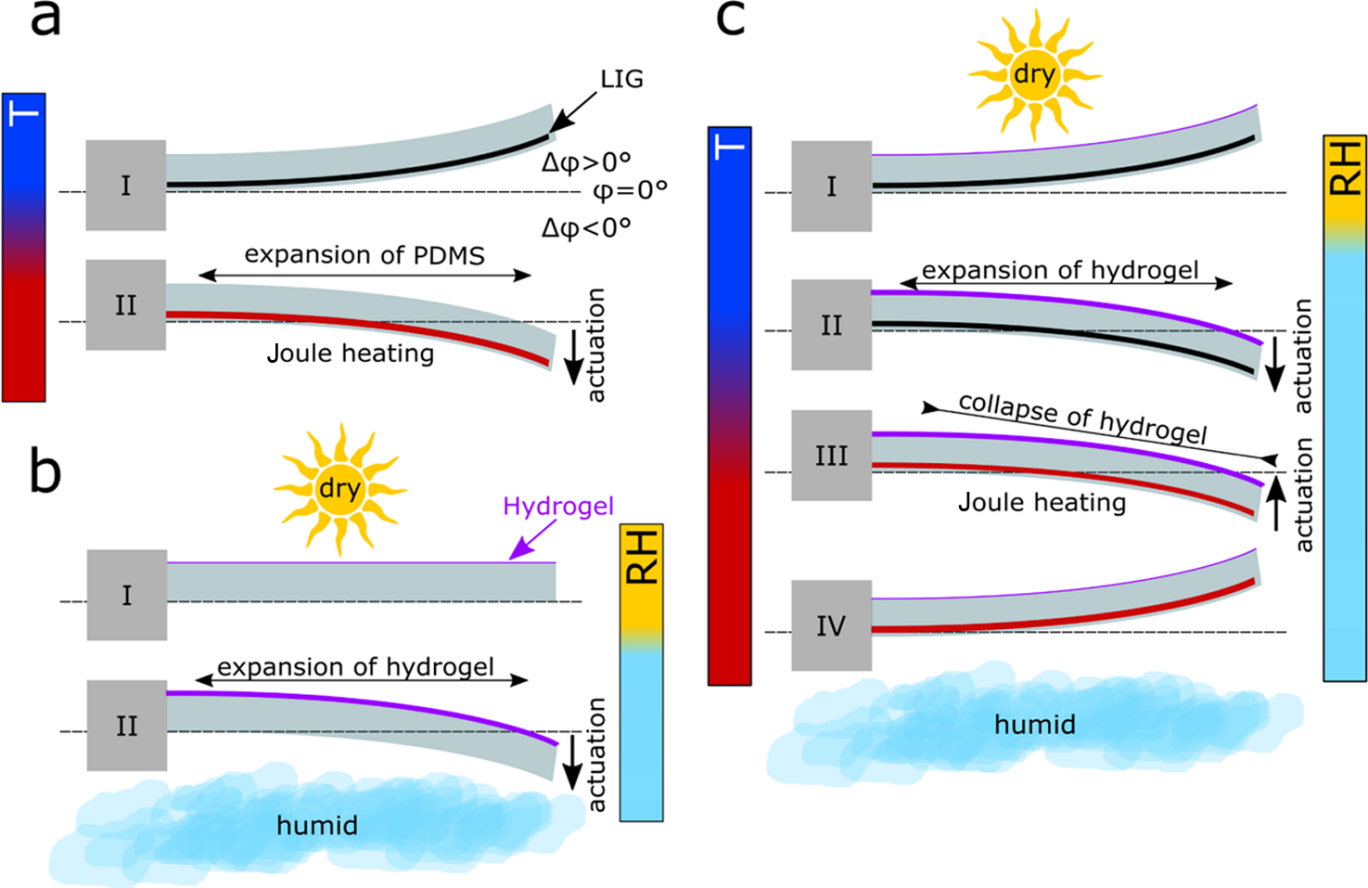
Schematic actuation mechanism for different types of actuators: (a) thermoresponsive actuator (PDMS/LIG) in initial (I) and actuated positions (II); (b) humidity-responsive actuator (PDMS/pNVCL) in initial (I) and actuated positions (II); and (c) multiresponsive actuator (PDMS/LIG/pNVCL) in the initial position (I), humidity-driven actuation (II), starting of Joule heating and collapse of the hydrogel (III), and back in the initial position at high humidity (IV).

Figure 3 shows the schematic actuation mechanism for the different actuation types. The characteristics of the thermoresponsive PDMS/LIG actuator (Figure 3a), the humidity-responsive PDMS/pNVCL actuator (Figure 3b), and the multiresponsive PDMS/LIG/pNVCL actuator (Figure 3c) are evaluated and discussed in the following sections.

### 2.2 Thermoresponsive PDMS/LIG Actuator

**Figure 4:**
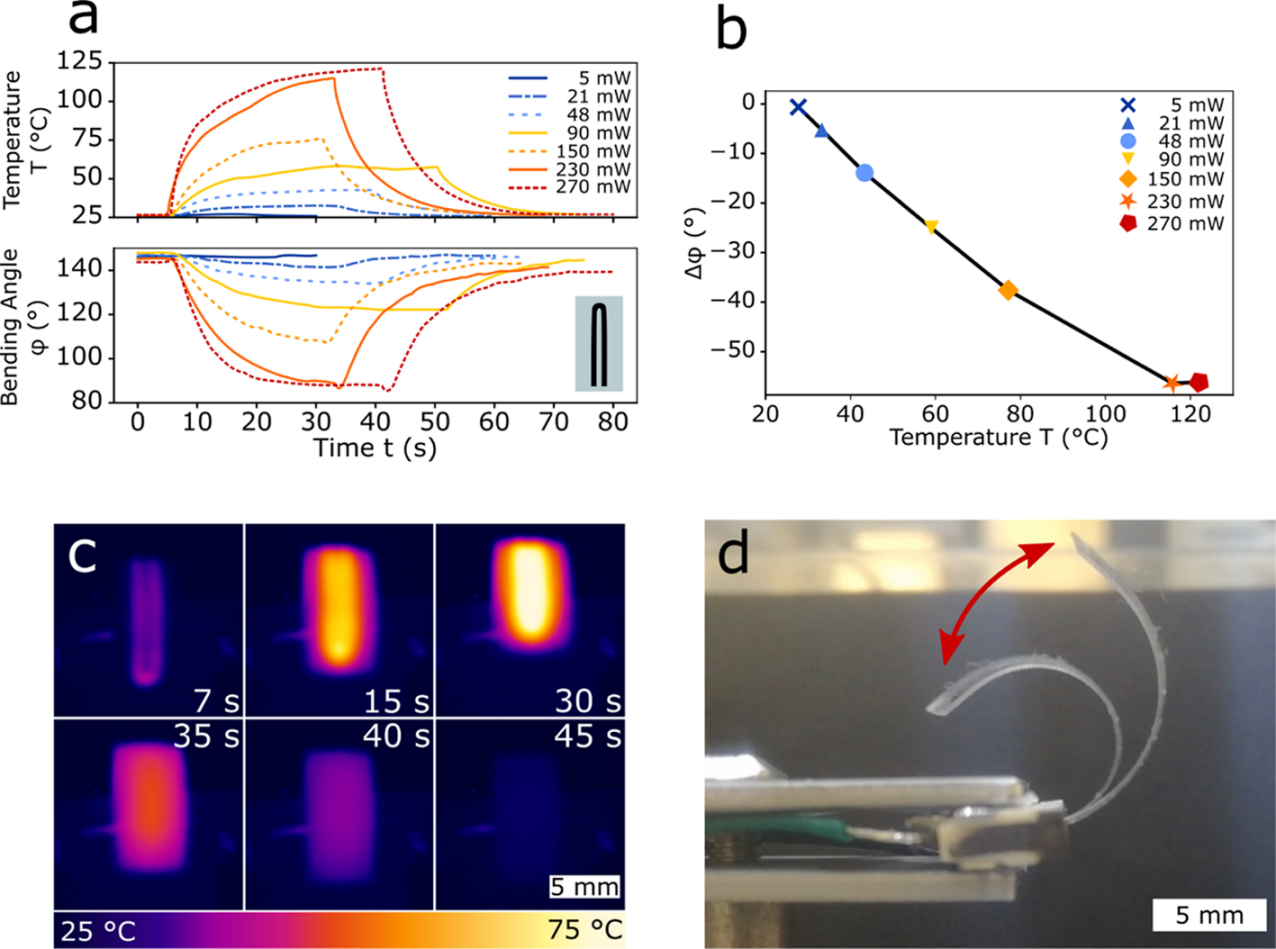
Thermoresponsive behavior of PDMS/LIG actuators with localized Joule heating. (a) Temperature *T* and corresponding bending angle φ over time for a PDMS/LIG actuator (inset: actuator design) for Joule heating at various levels of applied electrical power starting from the OFF state (no current applied); (b) change in bending angle Δφ versus induced temperature *T* (extracted from (a)); (c) series of IR images showing Joule heating over time when the current (*P* = 150 mW) is applied; and (d) superimposed images showing the actuation at *t* = 0 and 35 s for *P* = 230 mW.

The thermoresponsive PDMS/LIG actuator without a hydrogel layer was characterized (Figure 4), so that the dependency of Joule heating on the PDMS could be investigated. For this characterization, a single U-shaped LIG (two conductive tracks) embedded in PDMS was used as a Joule heating element (inset of Figures 4a and S4). The bending angle φ and the corresponding temperature of the actuator as a function of applied power are shown in Figure 4a. The extracted change in bending angle between OFF (i.e., w/o applied current, initial state φ_0_) and ON states (i.e., w/ applied current, φ_on_), Δφ = φ_on_ – φ_0_, versus the induced temperature is plotted in Figure 4b. The initial bending state φ_0_ = 150° arose from the thermal shrinkage during the curing of the PDMS at *T* = 80 °C,^23^ while the LIG prevented the shrinking on one side. This induced an initial bending angle of φ > 0°, as illustrated in Figure 3a-I. The elevated temperature induced by the Joule heating elements caused the PDMS to expand according to the linear coefficient of thermal expansion, which is around 280 ppm/K.^24^ On the other hand, LIG, with a very small linear coefficient of thermal expansion similar to GO (0.85 ppm/K),^25^ prevented the expansion on the side where it was embedded. This resulted in a negative bending upon actuation (Δφ < 0°), as annotated in Figure 3a-II. Higher temperatures resulted in a larger expansion and therefore a larger (negative) bending. The temperature/actuation was limited by the combination of the power supply maximum voltage and the resistance of the heating tracks. The thermal camera images in Figure 4c show the LIG heating elements while the current (*P* = 150 mW) was turned on at 0 < *t* < 30 s (first row) and after the current was turned off at 30 < *t* < 45 s (second row). At *t* = 7 s, the LIG tracks used for heating were identified; at *t* = 15 s, the actuator was already homogeneously heated, while at *t* = 30 s, the maximum steady temperature (*T* = 75 °C) and actuation were reached. Starting at *t* = 35 s, the current was turned off again and the actuator started to cool down. At *t* = 45 s, the actuator cooled down to room temperature again (*T* = 25 °C) while coming back to the initial bending state. The actuation response time was limited by the thermal transport while heating and by thermal dissipation while cooling. A superimposed image of the actuator at different times (*t* = 0, 35 s, corresponding, respectively, to *T* = 25, 110 °C) showing the related bending angles (φ = 145, 88 °C) is provided in Figure 4d; the corresponding video can be found in Supporting Information Video S1. The influence of humidity on PDMS and PDMS/LIG actuators was checked by placing them in a humidity chamber which resulted in no actuation (Figures S5 and S6). This result agrees with the literature in that PDMS neither swells in liquid water nor is it sensitive to water vapor.^26^

### 2.3 Humidity-Responsive PDMS/pNVCL Actuator

**Figure 5:**
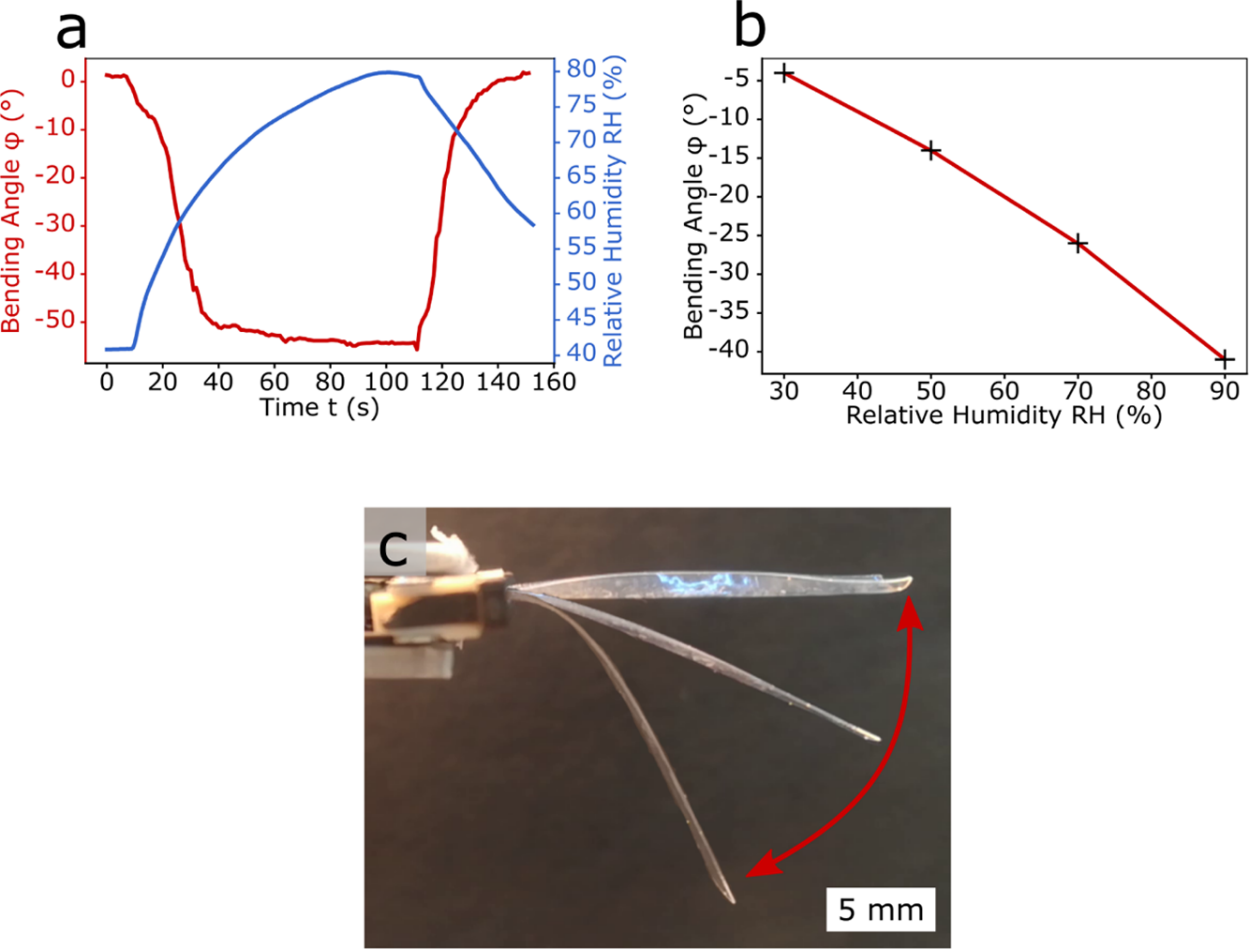
Humidity-responsive behavior of PDMS/pNVCL actuators. (a) Bending angle φ and humidity RH over time for a humidity-responsive PDMS/pNVCL actuator; (b) bending angle φ with relative humidity RH; and (c) superimposed images showing the soft actuator bending at *t* = 0, 25, and 80 s corresponding, respectively, to RH = 40, 55, and 80%, as reported in (a).

The humidity-responsive actuator was based on PDMS coated with pNVCL. No LIG heating elements were embedded in the PDMS, so that only the influence of humidity on the hydrogel and the bilayered composite could be investigated (Figure 5). According to the swelling curve (Figure 2d), the thickness of the hydrogel film increased with increasing humidity, while the PDMS had no response to humidity (Figure S5). This resulted in a negative bending actuation (Δφ < 0°) due to the swelling of the hydrogel, as annotated in Figure 3b-II and plotted in Figure 5a. It should be recalled that a thin pNVCL layer of only 300 nm induced enough force to bend the 500 times thicker PDMS matrix. The change in bending angle well corresponded to the change in humidity and was fully reversible, as shown in Figure 5a. It is important to note that due to the low thickness of the responsive hydrogel layer the actuation was faster than the response time of the humidity sensor, in agreement with the findings of Muralter et al. who used the same humidity sensor.^19^ The actuation time is around 20 s and is determined by the kinetics of the water intake/release by the hydrogel from the mist. The actuation time is comparable to or even better than those of other actuators in humid environments (6 min,^7^ 80 s,^27^ and 15 s^5^). In Figure 5b, the variation of the bending angle φ as a function of relative humidity RH is plotted. The discrepancy in the bending angle between Figure 5a,b comes from the different measurement conditions and instruments. While in Figure 5a the humidity level slowly increased (due to the high response time of the sensor and the chamber volume), the actual humidity in the chamber was already higher (near RH = 100%). In contrast, the humidity value in Figure 5b was set to RH = 90% and the measurement was done after a soaking time of around 5 min. Some superimposed images taken at different times (at *t* = 0, 25, 80 s) from the recorded video (Video S2, Supporting Information) are shown in Figure 5c. The repeated actuation of 100 times (Figure S7, Supporting Information) performed on a sample, which was stored for 1 year at room temperature, showed the same bending behavior and amplitude as the pristine one recorded 1 year before, just after preparation. This suggests that no degradation of the PDMS/pNVCL composite occurred during storing and repeated exercise. Additionally, no delamination of the active hydrogel layer from the supporting PDMS has been observed in any sample, not even in the abovementioned case of prolonged use or storage.

### 2.4 Multiresponsive PDMS/LIG/pNVCL Actuator

**Figure 6:**
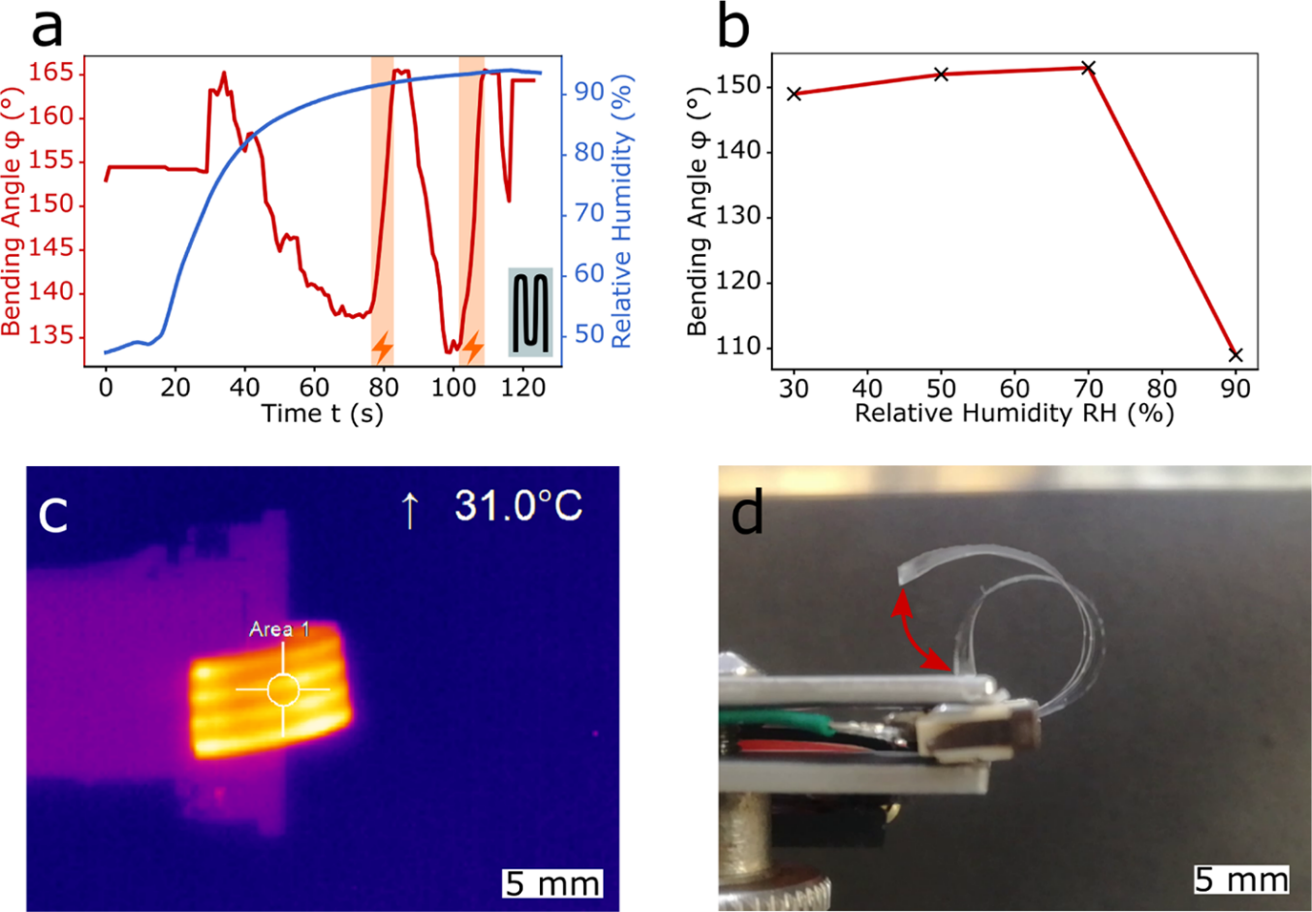
Multiresponsive behavior of PDMS/LIG/pNVCL actuators. (a) Bending angle φ and relative humidity RH plotted versus time for a multiresponsive PDMS/LIG/pNVCL actuator; orange areas indicate the ON state for applied current with a power of *P* ≈ 30 mW (inset: actuator design); (b) bending angle φ with relative humidity RH; (c) infrared image of the heated actuator (*P* ≈ 30 mW); and (d) superimposed image showing the actuation: φ = 135° with Joule heating and φ = 165° without Joule heating, both at RH ≈ 100%.

At last, both types of actuators were combined to create a multiresponsive actuator (Figure 6). As described before, the LCST of the hydrogel could be tuned by changing the deposition parameters and the cross-linker fraction. Taking into account that the measurements showed that both the thermoresponsive actuation (Figure 4b) and the hydrogel actuation were acting in the same direction (negative actuation Δφ < 0°, see Figure 3a-II,3b-II), the LCST should be set as low as possible. An LCST of 35 °C was chosen because at this temperature the counteracting force of the expanding PDMS is lower compared to the force induced by the hydrogel collapse and it is well above the room temperature. The recorded values for the bending angle and humidity are plotted versus time in Figure 6a. During the first 30 s, the actuator was resting on the sample holder until the humidity was high enough to counteract the intrinsic initial bending (*t* = 35 s). After this point, the swelling of the hydrogel resulted in a negative actuation (Δφ < 0°), as illustrated in Figure 3c-II. At around *t* = 80 s (first orange area), the Joule heating element was turned on and the actuator was heated up to around *T* = 30 °C (Figure 6c). The rise in temperature at around the LCST induced a collapse of the hydrogel, which resulted in a positive actuation (Δφ > 0°), as shown in the actuation schematic (Figure 3c-III). The rather late actuation response to humidity, also seen in Figure 6b, was because the expanding hydrogel first had to counteract the “preloaded” intrinsic bending while the actuator was resting on the sample holder. At a value of RH = 70%, the hydrogel expanded enough to lift the actuator further than the resting position. Comparing Figures 6c and 4c, it is important to note that for the multiresponsive actuator, a four-track (M-shape) heating element was scribed (see the inset of Figures 6a and S4). Since a low LCST was set and the results in Figure 4b showed that a temperature of around *T* = 30 °C prevented a large expansion of the PDMS, a heating element with a higher resistance (more tracks) could be used for a more uniform heating of the actuator. Due to the small dimension of the LIG tracks, the mechanical properties of the actuator were not changed and the thin pNVCL layer was still able to bend the matrix. In Figure 6d, superimposed images demonstrating the actuation range are shown (φ = 135° with heating and φ = 165° without heating, both at RH ≈ 100%). These were extracted from the recorded video that can be found in Supporting Information Video S3.

### 2.5 Self-Sensing and a Demonstrator

**Figure 7:**
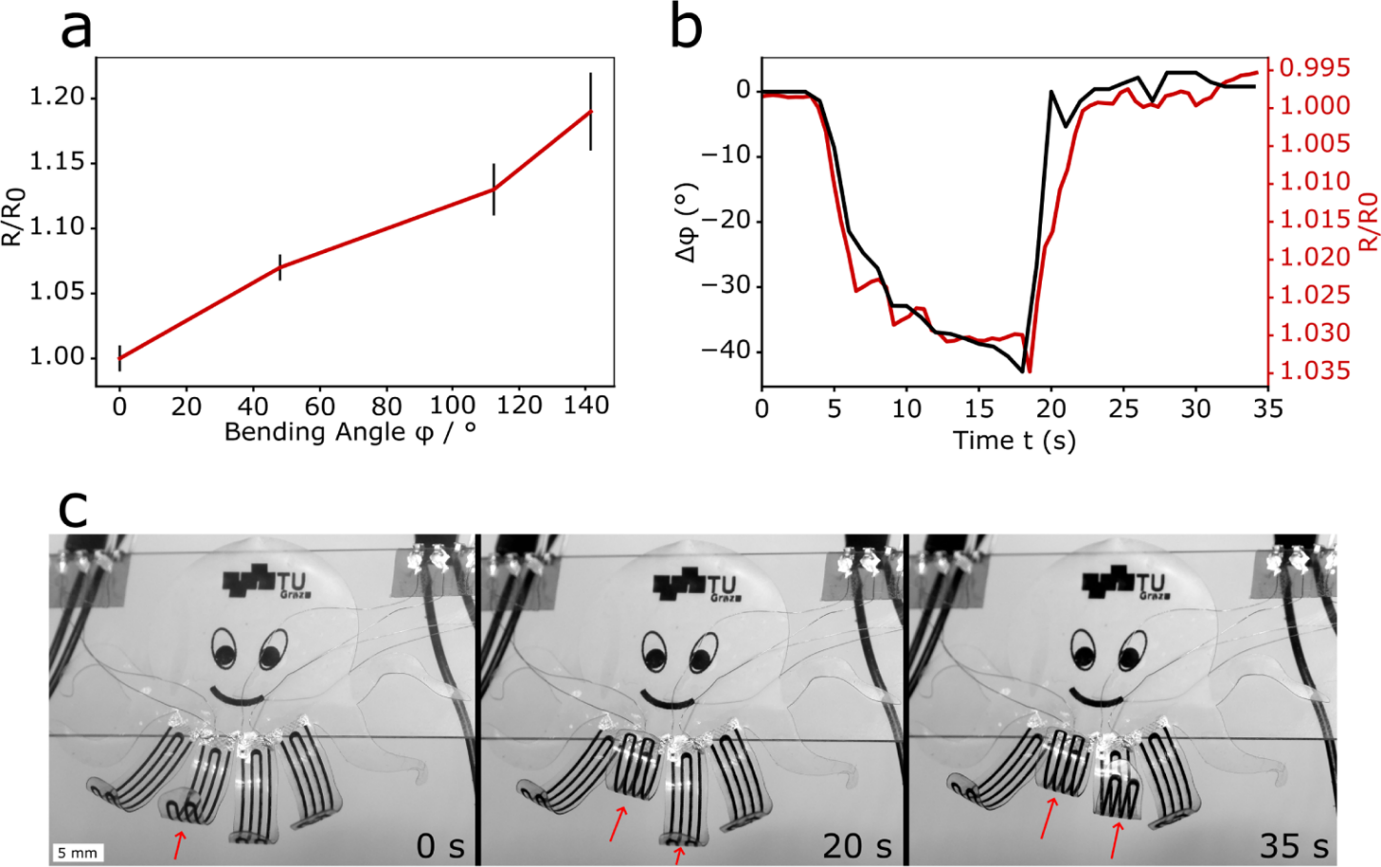
Piezoresistive behavior and self-sensing capabilities of PDMS/LIG/pNVCL. (a) Piezoresistive change induced by an imposed change in the bending angle with electromechanical tensile/compression testing equipment; (b) demonstration of self-sensing capabilities of multiresponsive PDMS/LIG/pNVCL actuators showing a correspondence of change in resistance (*R*/*R*_0_) and bending angle Δφ; and (c) series of three images showing the demonstrator with four actuator arms, which can be controlled individually.

Real applications of actuators also require feedback on the actuator movement. The embedded LIG could not only be used for Joule heating but also enabled the actuator to be self-sensing through its piezoresistive response, which we highlighted recently.^20^ When LIG is embedded in a matrix, as in the case of our actuators, a piezoresistive response can be observed.^20,28−31^ The main mechanism responsible for the piezoresistivity is a (partly) reversible crack formation in the embedded LIG due to imposed strain. A tensile testing setup (described elsewhere^20^) was used to induce a bending in the actuators by compressing them with a negative strain (see Figure S8 in the Supporting Information). In the first row of Figure 7, measurements regarding the self-sensing capabilities of the PDMS/LIG/pNVCL actuator are shown. Figure 7a shows the change in relative resistance (*R*/*R*_0_) depending on the induced bending angle φ. As φ gets larger, the LIG is exposed to more strain and the resistance gets larger due to the piezoresistivity. The piezoresistive response of the PDMS/LIG composite under positive strain (0–10%) can be found in Figure S9 in the Supporting Information. This mechanism was used to monitor the change in the bending angle φ, as seen in Figure 7b. The changes in resistance (*R*/*R*_0_) and bending angle Δφ of the PDMS/LIG/pNVCL actuator were measured while the actuator was exposed to a change from a dry to a humid environment and back. This resulted in a reversible actuation of Δφ = 40° and a reversible resistance change of 3.5%. The piezoresistive feedback of the embedded LIG could be correlated with the change in the bending angle Δφ and made it possible to track the actuator movement without any other measurement devices. It should be noted that this mechanism cannot be used in the case of a temperature-induced actuation because of the thermistor behavior of the LIG.^32^ The effect of humidity on the LIG was measured (Figure S10) and showed only a small effect.

To demonstrate the versatility of our approach, an octopus with four nonresponsive and four multiresponsive PDMS/LIG/pNVCL actuator arms was designed and assembled. The octopus was made in one go and with the same fabrication process as the multiresponsive actuator. The demonstrator was placed in an environment chamber at RH = 100%. The swelling of the hydrogel in a humid environment extended the four responsive arms of the octopus. By applying a current to each of the two central arms of the octopus, they were retracted separately. Figure 7c shows a series of three images taken during the experiment, showing the individual control of two feet. A video of the retracting feet can be found in the Supporting Information (Video S4).

## 3 Conclusions

In this study, soft actuators made from a new variant of LIG, PDMS, and a smart hydrogel, pNVCL, were assembled and investigated. For manufacturing, state-of-the-art technologies such as laser scribing and iCVD were used. Laser-assisted photothermal synthesis of LIG allowed a localized patterning with high speed, and good spatial control and resolution over the conversion of an insulating into a conductive material without the need for any masking or wet processing in ambient air. The iCVD allowed uniquely combining the following advantages: (i) the hydrogel layer had a uniform small thickness of 300 nm; (ii) notwithstanding the low thickness, the hydrogel showed a high humidity response due to the retention of the monomer chemical functionalities during the polymerization; (iii) the deposition neither damaged the PDMS nor the LIG/PDMS system; indeed, no bending was observed upon the iCVD treatment. The actuation mechanism of each type of materials system was studied. PDMS/LIG actuated depending on the temperature; PDMS/pNVCL showed a humidity-responsive actuation instead. The combination of the first two, PDMS/LIG/pNVCL, led to a multiresponsive actuator. It was shown that the embedded LIG tracks could also be used to self-sense the change in bending angle by measuring their piezoresistive change. To show the versatility of this approach and to demonstrate the independent control of each actuator, an octopus with four multiresponsive arms was designed. Single arms could be changed from an extended state to a retracted state independently and repeatedly. The actuation response obtained by our soft actuators with just a 300 nm thin hydrogel active layer can enable several applications of these systems, for example, in MEM devices, artificial muscles, and soft and biomimetic robotics.

## 4 Experimental Section

### 4.1 Laser Scribing Parameters

A laser cutter/engraver (Universal Laser Systems VLS 2.30, Power 30 W) operating with a CO_2_ laser source at 10.6 μm wavelength and equipped with an HPDFO beam collimator (nominal beam size: 30 μm) was used to create conductive patterns of LIG onto PI tape (Kapton HN, thickness = 50 μm, with silicone glue, supplied by RS Components Handelsgesellschaft m.b.H.). The laser processing for LIG production was operated in ambient conditions, with the PI tape attached onto a 1 mm thick glass slab. The laser cutter was operated in a raster mode through its native software (Universal Laser System Interface). The following laser rastering parameters were employed for producing an LIG variant, which is lifted off from the surface (LIG-LO): power = 11%, speed = 17%, raster resolution of 1000 PPI, image density (ID) of 7 (arbitrary scale, defining a spacing between consecutive rastered lines of ≈30 μm), and a positive defocusing of 0.7 mm. The corresponding laser fluence H was calculated as described in the Supporting Information. Additional settings for cutting and lasering the holes in PDMS for the electrical contact can be found in Supporting Information Table S2.

### 4.2 Spin Coating

The SYLGARD 184 PDMS prepolymer and curing agent were manually mixed at an 8:1 ratio and degassed. The scribed PI sheets were then spin-coated with PDMS at 750 rpm for 60 s, with a Chemat Technology KW-4A spin coater. After an initial curing at 80 °C for 10 min, another layer of PDMS was spin-coated with identical parameters and completely cured at the same temperature for 2 h.

### 4.3 iCVD Conditions

The hydrogel film on top of the PDMS was fabricated in a custom-built iCVD reactor described elsewhere.^33^ The chemical components used in the deposition process were *N*-vinylcaprolactam (NVCL, 98%, Merck, Germany), di(ethylene glycol) divinyl ether (DEGDVE, 99%, Merck, Germany), and *tert*-butyl peroxide (TBPO, 98%, Merck, Germany). To enhance the saturation pressure of both NVCL and DEGDVE, their jar heating was set to 85 and 75 °C, respectively. The flow rates were regulated by needle valves and set to (0.20 ± 0.05) sccm for NVCL, (0.35 ± 0.15) sccm for DEGDVE, and (1.02 ± 0.05) sccm for TBPO. During the polymerization process, the substrate was cooled to 35 °C and the filament temperature was set to 200 °C while maintaining a working pressure of 350 mTorr.

### 4.4 Manufacturing of Actuators

The actuator design was drawn using computer-aided design (CAD) software and transferred to the laser cutter. The PI (PI tape mounted on glass slides) was turned into LIG with the aforementioned laser scribing parameters according to the selected design (a U-shape or an M-shape, see the Supporting Information). The PI with the scribed LIG features was then spin-coated with PDMS and cured. pNVCL was then deposited onto the PDMS side in iCVD chamber. The actuator samples were then cut to shape with a laser cutter, and holes for the electrical connection were lasered. For stress-free peeling, the PDMS/LIG composite samples (still supported on the PI + glass slab) were put into an ethyl acetate bath; this resulted in a swelling of the PDMS and detachment from the PI substrate. After rinsing the samples with distilled water, the lasered holes were filled with stretchable silver ink (Engineered Conductive Materials, highly conductive, highly flexible silver ink, CI-1036) and then dried in an oven at 80 °C for 15 min for providing external interconnections for wiring.

### 4.5 Characterization

The thickness of the spin-coated PDMS film was measured by monitoring the cross sections under a Leica Wild M3B optical microscope. The mechanical properties were investigated with a custom tensile testing setup already described elsewhere.^20^ The same tensile testing setup was used to induce the bending angles for the self-sensing measurements. Certain negative strain values were set, and the resistance change was recorded. The bending angle was then extracted from images taken during the measurement. The LIG morphology was investigated with a JEOL JSM-6490LV scanning electron microscope operating at 10 kV acceleration voltage. The Raman spectra were measured using a LabRam HR800 combined with an Olympus BX41 microscope with a laser at a wavelength of 352 nm (5 mW). The spectra shown are the average of several spectra taken at different positions on the sample; they have been background-corrected, and the intensity of the G band has been normalized. A J.A. Woollam ESM-300 spectroscopic ellipsometer was used to determine the optical properties of the fabricated pNVCL thin films and their thickness. A three-layer model was used to fit the generated data, in which one layer represented the silicon substrate, another layer was the native silicon dioxide, and the hydrogel layer was modeled by a Cauchy function. The change of the thickness of the hydrogel with temperature, while being immersed in deionized water, was measured in a J.A. Woollam heated liquid cell. A temperature ramp of 0.5 °C/min was used to heat the hydrogel up to 50 °C. CompleteEASE software provides a feature that takes the surrounding water and its change of optical properties, depending on the cell’s temperature, into account. Measurements of the thermal stability and the response to humidity were performed with a Linkam THMSEL600 variable temperature stage.

### 4.6 Bending Experiments

Measurements were carried out in a custom humidity chamber with a small water atomizer and an SHT15 humidity sensor plus Arduino. An Espec SH-222 temperature/humidity chamber was used for the static measurements. A custom holder made from a zero-insertion force (ZIF) connector was used to mount the samples horizontally and connect them electrically to a Keithley 2601B source meter. The latter was used to deliver controlled current to LIG tracks for localized Joule heating at various levels of applied electrical power. The actuation responses of the samples were recorded with a camera setup and examined by extracting single frames from the videos. These frames were used to measure the bending angles and assemble the superimposed figures. The temperature was measured with an Optris PI160 infrared camera, and videos were recorded and evaluated with “PI connect” software. A Keithley 2601B source meter sourcing 0.5 mA was used for the self-sensing measurements. The tensile test setup explained by Dallinger et al.^20^ was used for the induced bending, and the bending angle was extracted from pictures.

## Supporting Information Available

Exact Raman spectra band parameters; thickness and refractive index of pNVCL at different humidity levels over time; thermal stability of pNVCL; stress–strain curve of the PDMS/LIG composite; schematic of PDMS/LIG heating elements; influence of humidity on PDMS and PDMS/LIG; long-term bending stability (PDF)
Thermoresponsive PDMS/LIG actuator (MP4)
Humidity-responsive PDMS/pNVCL actuator (MP4)
Multiresponsive PDMS/LIG/pNVCL actuator (MP4)
Demonstrator showing individual control of single feet (MP4)
