# Supplementary material for: Multiresponsive Soft Actuators Based on a Thermoresponsive Hydrogel and Embedded Laser-Induced Graphene
Source: ACS Appl Polym Mater. 2021 Mar 9;3(4):1809–18. doi: 10.1021/acsapm.0c01385 (PMC8042638; doi:10.1021/acsapm.0c01385)
Supplement: Supplementary file 1 — ap0c01385_si_001.pdf [file ap0c01385_si_001.pdf]

Supporting Information

Multiresponsive Soft Actuators based on Thermoresponsive Hydrogel and embedded Laser-Induced Graphene

Alexander Dallinger[a], Paul Kindlhofer[a], Francesco Greco[a,+], Anna Maria Coclite[a,*]

[a] Institute of Solid State Physics, NAWI Graz, Graz University of Technology, Petersgasse 16, 8010 Graz, Austria.

* Corresponding author 1, email: anna.coclite@tugraz.at
[+] Corresponding author 2, email: francesco.greco@tugraz.at

Table S1: Summary and comparison of Raman spectra band parameters.

	LIG-Lift off	LIG-F [1]	LIG-P [1]
G-band position/cm^{-1}	1583.0	1587.3	1582.0
G-band width/cm^{-1}	68.1	56.2	55.4
G-band intensity/counts	80.2	98.8	99.4
D-band position/cm^{-1}	1338.9	1344.6	1342.6
D-band width/cm^{-1}	91.6	77.7	57.8
D-band intensity/counts	89.2	85.4	105.1
D/G-ratio	1.11	0.86	1.06

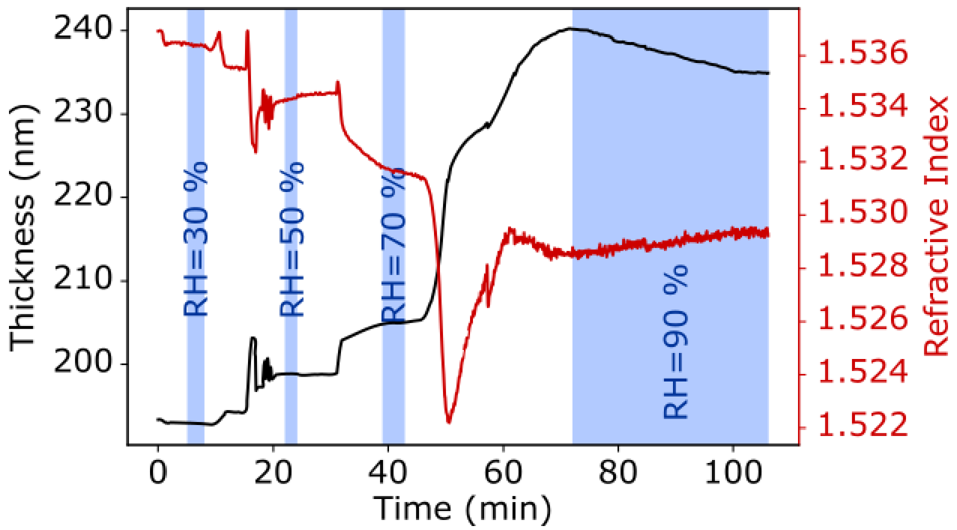

Figure S1: Thickness and refractive index of the pNVCL film at different humidity (blue areas) settings.

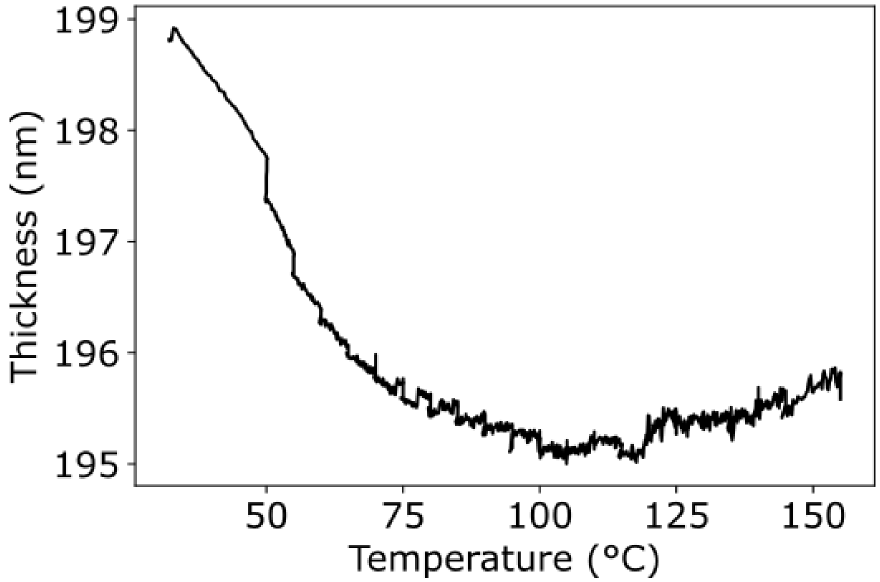

Figure S2: Thickness versus temperature for assessment of pNVCL hydrogel thermal stability.

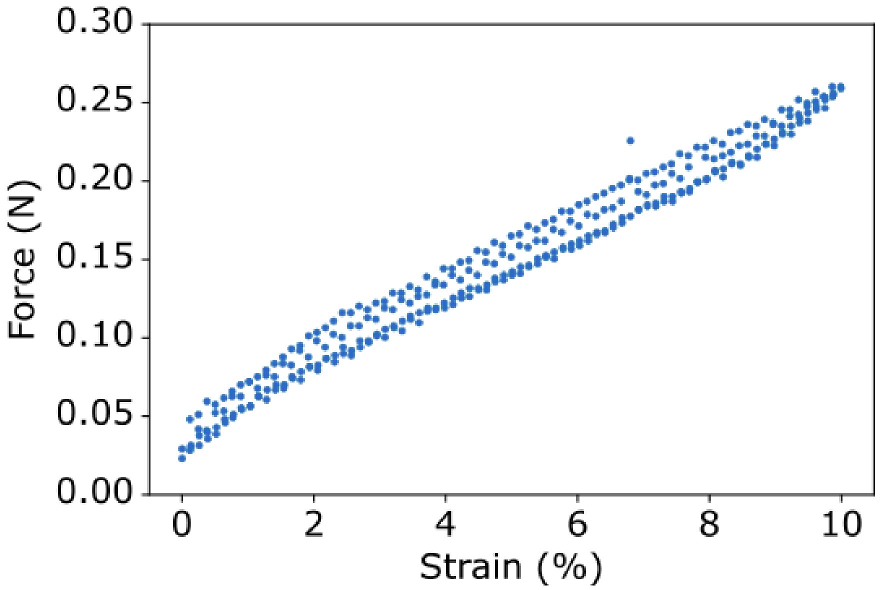

Figure S3: Force curve of PDMS/LIG composite with a strain cycle of 10% imposed 5 times.

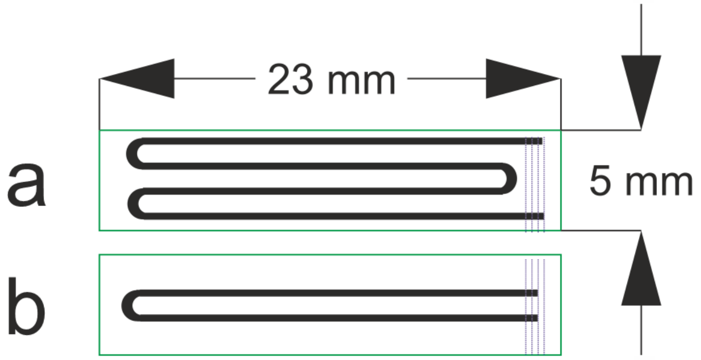

Figure S4: Schematics of heating element designs used for actuation, a) M-shape for multiresponsive actuation, b) U-shape for thermoresponsive actuation.

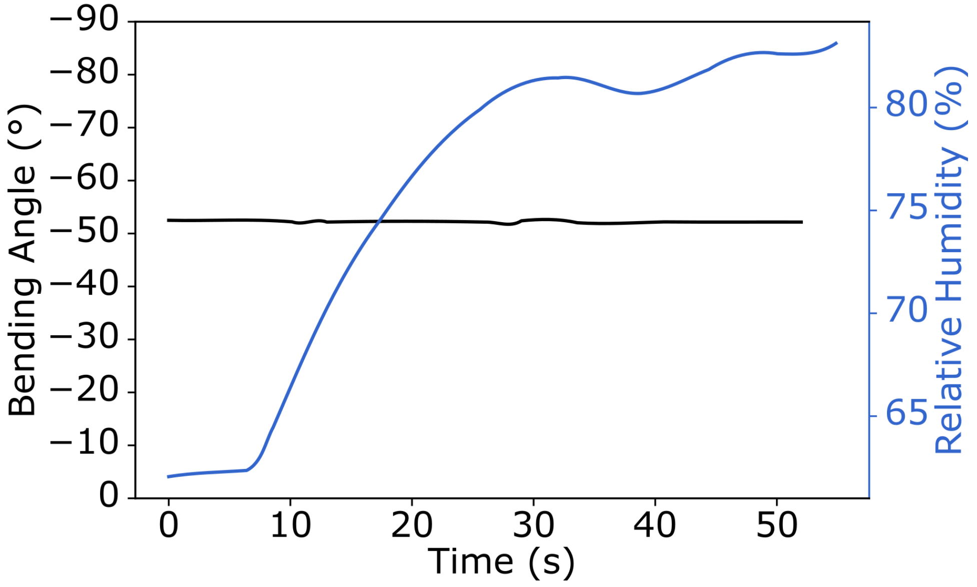

Figure S5: Bending angle and humidity curves for a PDMS sample showing no response to humidity.

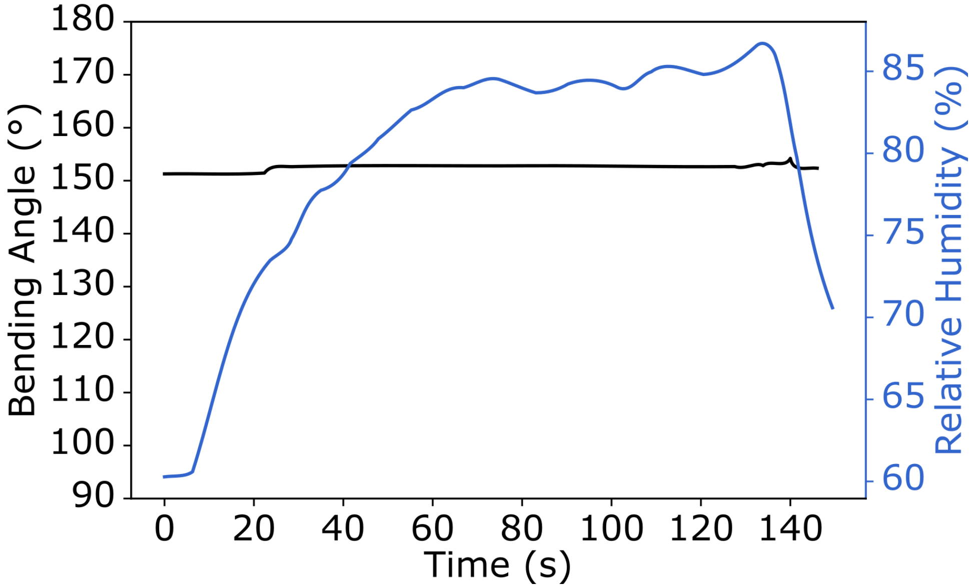

Figure S6: Bending angle and humidity curves for a PDMS/LIG actuator showing no response to humidity.

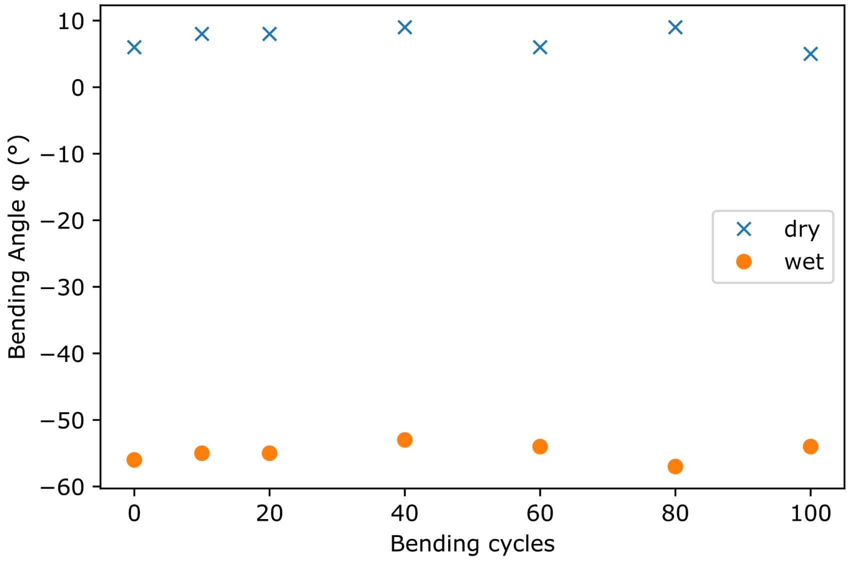

Figure S7: Bending angle for dry (~25% RH) and wet (~90-100% RH) environment of a PDMS/LIG/pNVCL actuator (M-shape LIG pattern, see Figure S4), showing the stability and repeatability of the actuator over 100 cycles for an one year old sample. The bending range and values are identical with the measurements done with the pristine sample (see Figure 5a).

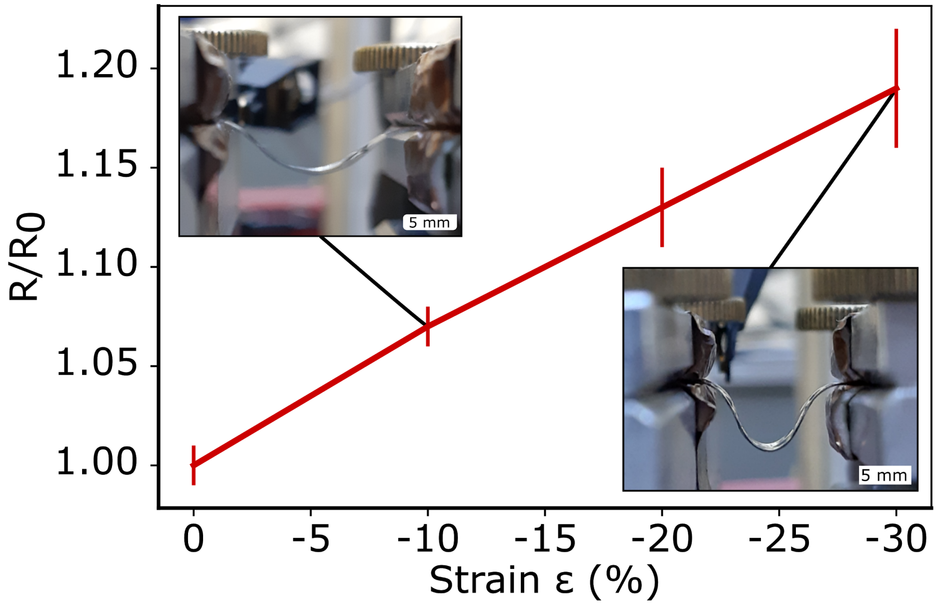

Figure S8: Electromechanical measurements showing the normalized resistance change R/R_0 for certain values of strain (ε = 10,15 and 30%). Insets show the induced bending which were extracted and used in Figure 7a.

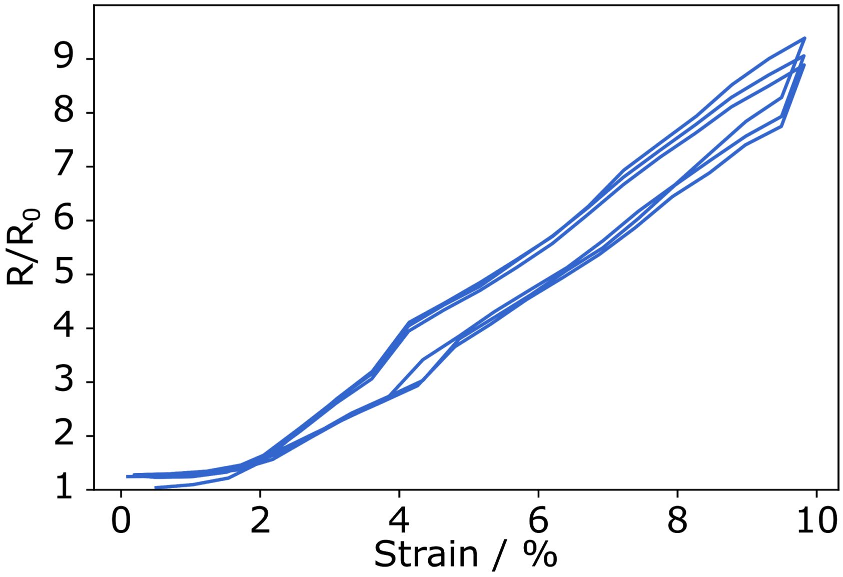

Figure S9: Electromechanical measurements under the repeated imposition of tensile strain showing the piezoresistive response of the PDMS/LIG composite plotted as relative resistance R/R_0. A positive strain was imposed three times with a tensile test setup [1] and the resistance change was recorded.

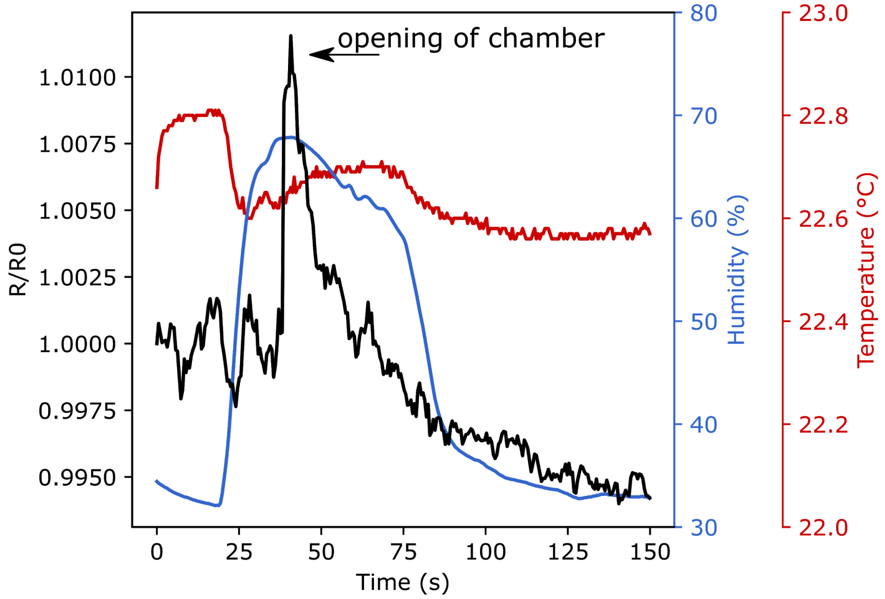

Figure S10: Effect of Humidity on the resistance of the LIG/PDMS composite used for self-sensing. The spike between 25 and 50 seconds shows the opening of the measurement chamber and is related to the movement of the actuator due to air movement.

Calculation of laser fluence

The laser fluence H for the laser raster setting was calculated as

$$H = \frac{P \cdot P_{max}/100}{s \cdot v \cdot PPI}$$

where P is the set laser power (%) with respect to the maximal laser power P_{max}= 30 W, s is the theoretical laser spot size determined according to gaussian beam theory, v is the measured processing speed and PPI the raster resolution.

Tabel S2: Laser parameters for laser cutting and LIG scribing at VLS 2.3.

	**Mode**	**Power**	**Speed**	**PPI**	**z**	**ID**
LIG scribing	raster	11%	17%	500	0.7	7
PDMS cutting	vector	57%	96%	500	0.7	5
PDMS holes	raster	43%	10%	500	0.7	5

References

1. Dallinger, A.; Keller, K.; Fitzek, H.; Greco, F. Stretchable and Skin-Conformable Conductors Based on Polyurethane/Laser-Induced Graphene. *ACS Appl. Mater. Interfaces* **2020**, *12* (17), 19855–19865. https://doi.org/10.1021/acsami.0c03148.
